# Single Cell Gene Expression to Understand the Dynamic Architecture of the Heart

**DOI:** 10.3389/fcvm.2018.00167

**Published:** 2018-11-21

**Authors:** Andrea Massaia, Patricia Chaves, Sara Samari, Ricardo Júdice Miragaia, Kerstin Meyer, Sarah Amalia Teichmann, Michela Noseda

**Affiliations:** ^1^British Heart Foundation Centre of Research Excellence and British Heart Foundation Centre for Regenerative Medicine, National Heart and Lung Institute, Imperial College London, London, United Kingdom; ^2^Wellcome Trust Sanger Institute, Wellcome Trust Genome Campus, Cambridge, United Kingdom

**Keywords:** heart, gene expression, single cell, cellular landscape, transcriptomics, qRT-PCR, RNA-seq

## Abstract

The recent development of single cell gene expression technologies, and especially single cell transcriptomics, have revolutionized the way biologists and clinicians investigate organs and organisms, allowing an unprecedented level of resolution to the description of cell demographics in both healthy and diseased states. Single cell transcriptomics provide information on prevalence, heterogeneity, and gene co-expression at the individual cell level. This enables a cell-centric outlook to define intracellular gene regulatory networks and to bridge toward the definition of intercellular pathways otherwise masked in bulk analysis. The technologies have developed at a fast pace producing a multitude of different approaches, with several alternatives to choose from at any step, including single cell isolation and capturing, lysis, RNA reverse transcription and cDNA amplification, library preparation, sequencing, and computational analyses. Here, we provide guidelines for the experimental design of single cell RNA sequencing experiments, exploring the current options for the crucial steps. Furthermore, we provide a complete overview of the typical data analysis workflow, from handling the raw sequencing data to making biological inferences. Significantly, advancements in single cell transcriptomics have already contributed to outstanding exploratory and functional studies of cardiac development and disease models, as summarized in this review. In conclusion, we discuss achievable outcomes of single cell transcriptomics' applications in addressing unanswered questions and influencing future cardiac clinical applications.

## Introduction

Each single cell of our body has a unique position in space and time and is, therefore, exposed to a unique set of specific signals and stimuli. However, until recently, our capacity to study single cells was limited to handfuls of genes or proteins and the vast majority of gene expression studies were done in bulk. Single-cell gene expression analysis started with the development of methods to study targeted transcripts including quantitative RT-PCR (1–3) but also single-molecule FISH that allows to maintain the spatial information (4–7). Approximately a decade later, the first paper proving the feasibility of single cell RNA-sequencing (scRNA-seq) was published, evidencing its superiority to single cell microarrays in the study of single mouse blastomeres, with 75% more genes detected and a level of resolution permitting the identification of more than 1,700 previously unknown splice junctions (8). Thus, a new era started and a complete change of perspective, permitting the analysis of gene expression and, consequently, cell function and intercellular networks from a cell-centric viewpoint.

There has been rapid development of technologies to reduce noise, improve sensitivity and, notably the throughput for single cell transcriptomics (9–17). Indeed, the number of high-impact studies using scRNA-seq is rapidly growing in many fields. Pioneering studies were conducted on developing embryos, even at the pre-implantation stage, circulating cells and also solid tissue including human disease samples such as cancer (8, 9, 14, 18–23). The first single cell gene expression studies on the heart relied on single cell qRT-PCR and scRNA-seq studies lagged for a few years (24, 25). The initial work focusing on cardiac development as well as adult heart demonstrated feasibility with some useful explorative studies and more recent ones proving the unique value of single cell studies in identifying pathways' dysregulation in disease models (26–29).

The advantages of single cell gene expression analysis offsets any associated technical difficulty. Three fundamental features can be defined at the single cell level for the expression of each gene and, more relevantly for patterns of gene expression predicting a specific cell type: prevalence, heterogeneity, and co-expression. In particular, for single cell transcriptomics based on each cell's own global gene expression, the classification of cell types is uniquely quantitative and data-driven, allowing the discovery of markers defining a tissue or cell type without prior knowledge (17, 19, 30–38). In fact, the sensitivity and specificity of whole-transcriptome signatures is above technical noise and biological variability, and therefore is sufficient to discriminate novel and rare cell types and previously unappreciated markers during development, and disease progression (14, 17, 19, 22, 36–42).

The use of scRNA-seq to unravel heterogeneities, identify novel cell populations and organize cellular hierarchies, represents the first explorative phase enabling a dynamic vision with functional implications that would otherwise be masked in bulk analysis (17, 21, 36–39, 42–46). From a computational perspective, the elaboration of this rich information using unsupervised clustering reliably allows to group cells according to their subtype or state, as shown in experiments deconvoluting the cellular composition of complex tissues (brain, spleen, intestine, ear, retina, and tumors) but, also, to characterize transitions along pseudotemporal trajectories and reconstruct lineage decisions (37, 41, 47–51). Indeed, integrated data analysis entails subsequent deconstruction/reconstruction iterations to define the regulatory and signaling mechanism governing cellular decision that result in the definition of specific cell types and/or functional states. Remarkably, single cell transcriptomics allows the investigation of gene regulatory networks, which can be seen as the ensemble of active transcription factors and the genes they target (52), aiming to predict the effect of disruptions and manipulations to such network. In addition, previously masked by whole-tissue or pooled cell analysis, scRNA-seq also demonstrated the bimodal (on/off) distribution of gene expression in individual cells (22, 53). Ultimately, as described in more detail below, inferences go beyond the boundaries of single cells permitting the extrapolation of intercellular regulatory gene networks (38, 54–56).

In summary, single cell transcriptomics provides a global and unbiased view of tissues' cellular demographics from bottom up, both at steady-state and in dynamic processes like development, differentiation, and progression of disease. In the first section, we provide a detailed overview of the practicalities to design single cell RNA-seq or targeted gene expression experiments, and discuss methods including computational analysis; in the second part we cover the advancements brought by these new technologies in the cardiac field, the foreseeable successes in resolving ambiguities of heart biology and the potential applications in clinical settings.

## Experimental and computational approaches for single cell gene expression analysis

### Design of single cell transcriptomics experiments

**Figure 1:**
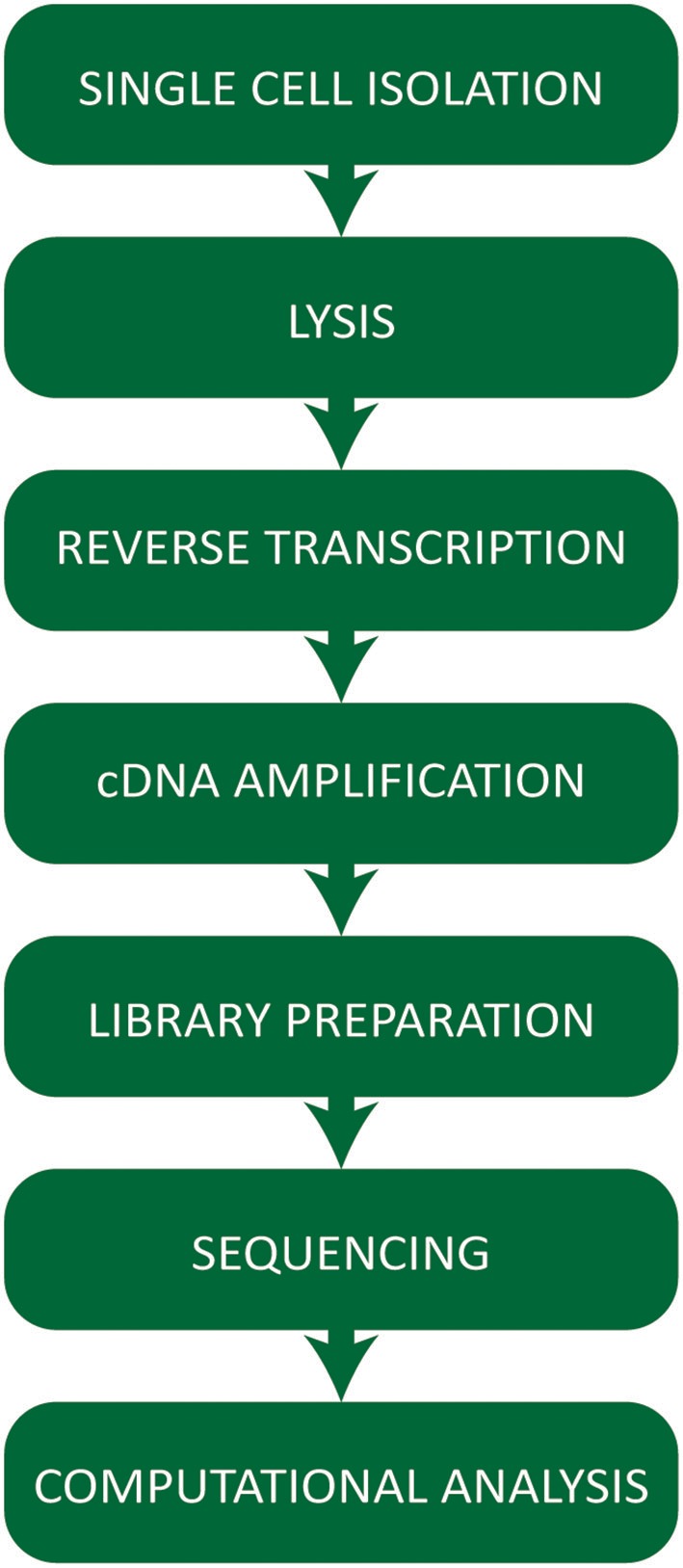
Workflow summarizing the critical steps of a typical single cell RNA-sequencing experiment.

Single cell RNA-seq technologies involve a number of steps (Figure 1) employing different strategies for cell capture, reverse transcription, cDNA amplification, and the increasing number of options available raises practical questions for first-time users.

The first decision concerns the number of cells to be captured and the transcriptome coverage needed, i.e., the number of genes detected per cell. The numbers required for an accurate representation of the reality will vary according to the specific biological context and the heterogeneity expected within the cell population under study. For example, if the objective is to capture all the cell types present in a tissue, it is advisable to consider methods that yield thousands of single cells, even with lower coverage per cell, because differences between cell-types involve so many genes that even a shallow characterization will be enough to distinguish them (57). On the other hand, if the focus of the study is the in-depth characterization of a specific cell-type that shows subtle differences in “state,” capturing more genes per cell can be more informative and accurate (57), even if only hundreds of cells are analyzed.

The number of cells that can be captured might also be conditioned by the available tissue size and/or the abundance of the cell types of interest. Approximate estimation of the number of cells required for a given experiment can be based on prior knowledge about the biological setting; alternatively, a more precise estimate of expression mean levels and dispersion as well as dropout rate can be obtained by specialized tools like powsimR (58), that predicts power and sample size requirements to detect differential expression by fitting a polynomial regression to data provided by the investigator, or applying user-generated parameters.

To choose the method for single cell isolation and capturing, the desired experimental outcome needs to be weighed against the availability and source of tissue and/or cells. Indeed, the source of cells, ranging from 2D cell cultures, to small cell aggregates, tissue sections, small fresh or frozen biopsies and large tissue samples, crucially determines the process to follow (Table 1). The availability of tissue sections and the need to preserve tissue structure and single cell localization, restricts the choice to laser capture microdissection (LCM) (59–61). This is a time consuming and very low throughput method, requiring staining to define cell boundaries and/or types which is useful to capture single cells in a specific micro-niche (62). Direct single cell trapping from the tissue was used in a variety of settings, including to study the unique identity of mouse and human motor neuronal populations based on their position along the spinal cord and most importantly to uncover the paracrine effects of mesenchymal stem cells (MSCs) on the myocardium of infarcted mouse hearts (63, 64).

**Table 1:** Comparison of the main features of commonly used single cell capturing techniques.

	**Plate-based (13, 16)**	**Valve-based microfluidics (65)**	**Droplet-based microfluidics (66)**	**LCM (63)**	**Micromanipulation (67)**	**ICELL8 (68)**
Need for dedicated equipment	No	Yes	Yes	Yes	No	Yes
Samples	Cells in suspension or dissociated	Cells in suspension or dissociated	Cells in suspension or dissociated	Tissue sections	Cells in suspension or dissociated	Cells in suspension or dissociated
Volume	Microliter	Nanoliter	Nanoliter	Nanoliter	Microliter	Nanoliter
Starting cells (minimum)	>10,000	Thousands	2,000-10,000	NA	Any	Hundreds
Number of cells captured	Hundreds	Hundreds	Thousands	Tens	Tens	Hundreds

*Need for dedicated equipment refers to the necessity for equipment exclusively designed for single cell RNA-seq capturing; samples refers to the source of single cells; volume indicates reaction size; starting cells reports the typical minimum number of cells required (NA, non applicable); number of cells captured refers to the typical number of events selected by the indicated technique*.

**Figure 2:**
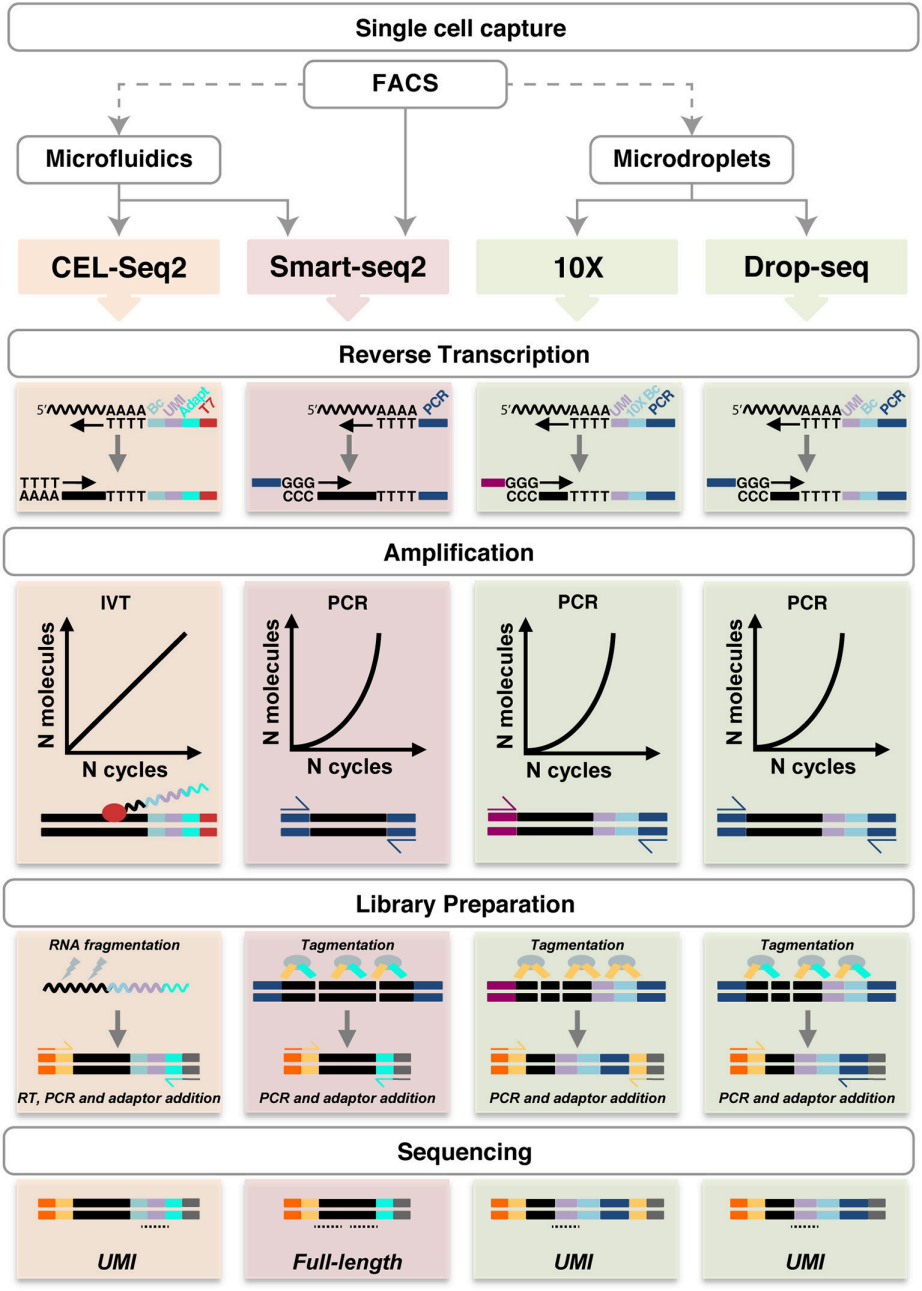
Schematic representation of single cell RNA sequencing experimental pipelines. FACS (with or without Index Sorting), microfluidics and microdroplets are the main methods used for single cell capture. Notably, FACS can be performed as a preparatory step before the other capture techniques. CEL-Seq2, Smart-seq2, 10X Genomics and Drop-seq are shown. Single cell capture is followed by lysis and reverse transcription using oligo-dT primers, which also introduce UMIs (lilac), barcodes (light blue), adapters (cyan), the T7 promoter (red) or PCR primers (dark blue and magenta), as shown in each specific method. Smart-seq does not include early barcoding. Second-strand synthesis in performed by poly(A) tailing (CEL-seq2) or by template-switching (in the other methods). The cDNA is then amplified linearly by IVT using the T7 promoter, or exponentially by PCR. During library preparation, the amplified molecules are fragmented by physical (CEL-Seq2) or enzymatic means (Smart-seq2, 10X and Drop-seq). Fragments are ligated with adaptor sequences required for cDNA amplification (yellow and cyan) and sequencing (orange and gray); in CEL-Seq2, this requires an initial RT step. The resulting sequencing libraries allow UMIs counting or full-length coverage. UMI, unique molecular identifier; Bc, barcode; IVT, *in vitro* transcription. Undulating lines represent RNA, solid blocks DNA, ovals enzymes, dotted lines sequencing reads. For more details, see https://teichlab.github.io/scg_lib_structs/.

All other techniques require a single cell suspension to be used as input. In this regard, it is important to consider that mechanical and/or enzymatic cell isolation can affect cell viability and potentially gene expression profiles, thus, exposure to enzymes (e.g., trypsin or collagenase) and higher temperatures should be minimized. A number of different methods are available to capture single cells (Table 1 and Figure 2).

Micromanipulation allows to manually pick single cells in suspension derived from culture or tissue using an inverted microscope and glass micro-pipettes (67, 69). Even if this method is time consuming, it can be useful to isolate single cells from samples with very few cells, such as early embryos or for large cells like CMs that cannot be unbiasedly selected by current flow sorters or most microfluidic apparatuses, and finally can be also used to select single nuclei (69). The purity of cells obtained will depend greatly on the operator.

The use of flow cytometry for cell capturing has the advantage of selecting and sorting single cells based on their expression of surface markers, fluorescent reporter proteins and/or fluorescent dyes defining their functional status (e.g., viability markers, cell cycle staining), allowing single cell multi-parametric, high throughput sorting into plates (e.g., followed by Smart-seq2) or in a tube for droplet-based methods, Massively parallel RNA single cell sequencing (MARS-Seq) (21), or virtually any other scRNA-seq application. Additionally, unique advantage of FACS is to perform index sorting, allowing the record of the fluorescence information of each parameter analyzed for each single sorted cell and to index it with the position of the sorted event. This enables the retrospective interrogation of flow cytometric parameters of unbiasedly sorted cells for which gene expression profiles has been acquired, providing a deeper understanding of the mechanisms involved in the function of that given cell, and potentially leading to the identification of new markers for populations of interest (70, 71). Importantly, FACS efficacy, accuracy and purity of >95% has been widely demonstrated (72, 73). The major limitation appears to be the relatively large amount of starting cells required (more than 10,000) and the size of sortable cells (19). Indeed, larger cells cannot be accurately and unbiasedly selected by FACS nor by most droplet-based methods. This is an important limitation for the study of single CMs, which reach a length of 150 μm in healthy hearts and even longer in certain disease states. A relatively new instrument, ICELL8, has the capacity to process cells of any size, although with medium throughput [up to 1,800 cells (68)]. The system is based on the use of a nano-dispenser that delivers cells to a chip containing 5,184 nanowells, each one preloaded with oligos which contain oligo-dT, barcodes and unique molecular identifiers (UMIs; as described in the next section); it integrates imaging to discriminate wells containing a single cell vs. multiplets and live/dead cells based on labeling with fluorescent dyes (68). Alternatively, large cells can be investigated by single nuclei RNA-seq (snRNA-seq), which was reported as sensitive and specific for the identification of CMs subtypes and an effective mean to profile expression dynamics in previously inaccessible frozen tissue (69, 74).

Additional approaches for capturing single cells are microfluidic-based devices and their combination with micro-droplets methods. Microfluidic systems enable sorting into individual compartments, and in the case of the valve-based Fluidigm C1, visual inspection is possible before further processing of the cells (12, 65). The device requires an input of minimum 1,000 cells with a throughput of 96 cells per chip and cell recovery can be low (67, 75).

The combination of microfluidics with micro-droplet methods (droplet-based microfluidics) offer even more advantages, such as lower sample consumption and contamination risks, ultimately, reducing volumes of reagents used and therefore costs (11, 76). Drop-seq was one of the first methods developed that enabled highly parallel analysis of individual cells by RNA-seq via encapsulation of cells in nanoliter droplets with DNA-barcoded beads allowing to analyse 44,808 cells from the retina and identify 39 transcriptionally unique cell types (77). Similarly, the indexing droplets (inDrop) method is based on capturing cells in barcoded nanoliter droplets which, in this case, contain a hydrogel carrying photocleavable combinatorially barcoded primers. The system was initially used to demonstrate a heterogeneous differentiation potential after leukemia inhibitory factor withdrawal in thousands of single mouse embryonic stem cells (78). Within this space, the 10X Genomics system is of recent development and is becoming a first choice for many researchers because of its flexible workflow and high-throughput (66). This micro-droplets system is based on the encapsulation of 500 to 20,000 single cells (or nuclei) thanks to their solution to generate nano-droplets. The process has a capturing efficiency of more than 50% thanks to single Poisson distribution loading and is significantly faster compared to inDrop or Drop-Seq allowing to capture eight samples within minutes with massive throughput results (66). One potential disadvantage is the risk of capturing cells' doublets (or multiplets), nevertheless, contamination's rates of the preparations can be empirically predicted mixing cells derived from different species or, in the absence of internal controls, a more challenging approach is to use computational methods (21, 22, 66, 77, 79). This system has been used in many areas of research demonstrating the power of single cell transcriptomic analysis, which goes well-beyond the mere cataloging of novel cell types. For instance, in a recent study of nearly 100,000 single cells from human lung cancer, 52 stromal cell subtypes were defined, including novel subpopulations hitherto considered to be homogeneous and for which novel functional roles were identified (30). These included fibroblasts expressing different collagen sets, immunomodulating endothelial cells, and disease associated changes in T-cell subtypes pointing to novel immunotherapy targets as well as new biomarkers (30).

In summary, decision-making for the best suited cell capturing platform requires the evaluation of four parameters: number of cells needed to answer a given biological question, transcriptome coverage needed, input of cells vs. cells' availability, cell size (67, 79).

### Reverse transcription and cDNA amplification: available options

After the isolation and lysis of single cells, scRNA-seq requires the conversion of their RNA into cDNA and its amplification to generate libraries with a signal above sequencing sensitivity threshold. Following single cell capturing and consequent cell lysis, virtually all protocols select poly-adenylated RNA and generate cDNA by using poly-(dT) primers (Figure 2). Smart-seq (switching mechanism at 5′-end of RNA template) does not include early barcoding. Thus, it requires each sample to be processed individually, but has the advantage of enabling full transcript sequencing obviating to coverage biases caused by incomplete reverse transcription that occurs using poly(A) tailings (8, 14, 31, 80) (Figure 2). In 10X Genomics, long fragments of DNA (>50 kb) are encapsulated in a droplet, where they are labeled with semi unique barcode for sequencing by Illumina technology (66). The barcode presence determines the relative spatial orientation of the tags, creating a map with linked reads, in order to combine information from several tags. Sequencing of this small material requires amplification that can be performed by either polymerase chain reaction (PCR) or *in vitro* transcription (IVT). PCR allows for improved sensitivity, but does not retain strand information (13, 14). The optimisation of Smart-seq2 from Smart-seq increased the sensitivity and library yield as well as reduced the costs and the duration of the process down to 2 days (13, 16). Drop-seq was shown to allow the analysis of 10,000 single cells within 12 h, being among the fastest and more cost-efficient, especially to analyse large number of cells (77, 79). The CEL-Seq method was the first to use IVT, obviating to the requirement for a template-switch step and related reduced efficiency (9). Second generation CEL-Seq2, with optimized primers, reagents, clean-up, and library preparation steps has higher sensitivity, lower hands-on time and costs as well as being adaptable to various platforms, including Fluidigm C1, Drop-seq and inDrop (20). The major limitation remains the lack of information on most instances of splicing because of the 3′-bias (20), which can be overcome barcoding individual cells and pooling them for a linear amplification using only one RT round (9, 20). Ensuring full-length coverage across mRNA is essential to analyse alternative splicing forms as well as identification of single-nucleotide polymorphisms, regardless of the orientation of amplification (at the 3′- or at the 5′-end) (14, 81). Ideally, the samples can be barcoded during RT reaction, such as in CEL-Seq, or during the following step of sequencing and library preparation (Smart-seq/Smart-seq2). In order to reduce noise created by duplets and improve efficiency in gene expression analysis, UMIs have been introduced as internal validation controls; thus, randomly barcoding each individual mRNA molecule during the reverse transcription reaction can be used to determine the absolute amount of mRNAs in a targeted single cell (12, 20, 22, 42, 77, 78).

The choice of the ideal method needs to take in consideration sensitivity, accuracy, precision, power, and efficiency of the cDNA conversion and its amplification plus the throughput of the libraries generated. Work was done to compare methods in parallel experimentally and computationally (79, 82). An integrated framework to analyse scRNA-seq methods performances was developed by comparing 15 protocols computationally from 28 published single cell studies and 4 protocols experimentally (82). Sensitivity in detection of gene expression, tested using spike-ins, was generally high and largely dependent on sequencing depth. Nevertheless, accuracy did not depend as much on sequencing depth and was lower in CEL-seq, and MARS-Seq, possibly because of higher performance variability of these methods (82). Importantly, endogenous RNA was shown to be much more efficiently captured and amplified than ERCC spike-in molecules with consequent underestimated measures of sensitivity (82). Obviating to the limitations of a comparison based on exogenous spike-ins, captured at lower efficiency than endogenous transcripts, a comparison based on endogenous mRNAs found Smart-seq2 to be the most sensitive and accurate method. Indeed, Smart-seq2 has a lower drop-out rate and detects a common set of over 10,000 genes in more cells compared to UMI-based methods. However, CEL-Seq2, Drop-seq, MARS-Seq, and SCRB-seq (single-cell RNA barcoding and sequencing) have lower amplification noise (79). Finally, as cost can be a factor in deciding which approach to choose, Drop-seq is more cost-efficient for transcriptome quantification of large cell numbers, while MARS-Seq, SCRB-seq, and Smart-seq2 have higher efficiency when analyzing fewer cells as shown by power simulations (79).

### A few considerations about sequencing depth

As mentioned above, sequencing depth is an equally important aspect of the design. Higher depth clearly allows a higher resolution in describing the cellular landscape under study based on single cell gene expression with an upper limit determined by the technology in use. Ultra-low coverage sequencing (<10,000 reads/cell) have been used to identify cell types in heterogeneous tissues, however, this number heavily relies on the diversity of the cellular landscape under investigation and finer distinctions and resolution of the genes set explaining variation require moderately shallow sequencing depth (50,000 reads/cell) (65). Recently, a comparison of different single cell RNA-seq technologies based on the detection of exogenous spike-ins, showed that sensitivity (the minimum number of detectable RNA molecules) is strongly affected by sequencing depth, with only a slight improvement after 10^6^ reads/cell and saturation at ~4.5 × 10^6^ reads/cell (82). These data suggest that one million reads per cell is a good target. On the other hand, accuracy (the correlation between known input molecules and quantified expression values) showed saturation at 250,000 reads/cell.

### Computational methods for single cell transcriptomic analysis

**Figure 3:**
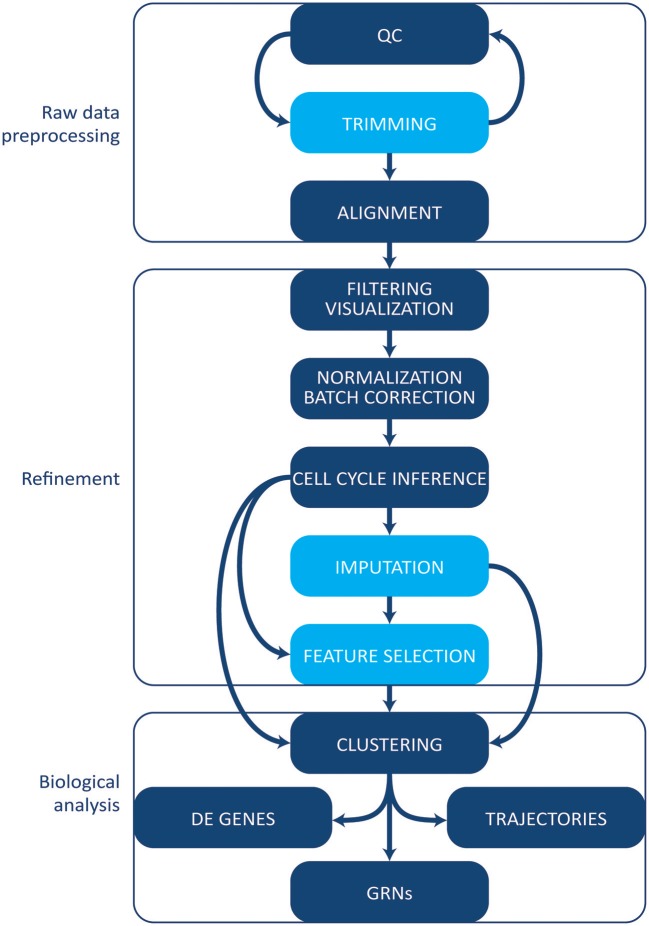
Computational analysis pipeline. The analysis can be broadly divided into three main stages (raw data processing, refinement and biological analysis), each including several steps. Initial QC and trimming can be performed iteratively, multiple times. Light blue boxes indicate optional steps. Details are given in the text. QC, quality control; DE, differentially expressed; GRNs, gene regulatory networks.

The increase in protocols and applications for single cell RNA-sequencing has been paralleled by the development of a multitude of computational tools and methods for single cell data. Some methods initially designed for bulk analyses can be applied to single cell experiments. However, single cell data tend to be noisier given the small amount of starting material, and several specific tools have been developed [reviewed in Stegle et al. (83)]. Here, we give an overview of the most common tools and methods employed in single cell transcriptomics' workflow analysis (Figure 3). We will not cover, however, tools and pipelines developed to be used in conjunction with proprietary technologies and systems (e.g., Cell Ranger by 10X Genomics or Singular by Fluidigm). Finally, we point the reader to the exhaustive archive at https://github.com/seandavi/awesome-single cell, which contains an exhaustive list of tools for single cells analysis, plus links to several in-depth tutorials, journal collections, and various resources.

### Raw sequencing data processing

Following demultiplexing, the earliest steps of the analysis pipeline (Figure 3) include assessing the quality of the data and preparing them for downstream analyses by filtering out low quality or contaminating reads and trimming adapters, primers, and low quality portions of the reads. Although trimming can be bypassed if the quality of the raw data is satisfactory, it generally allows for a faster and more accurate alignment.

Popular tools used at these stages include FastQC (https://www.bioinformatics.babraham.ac.uk/projects/fastqc/) for quality control and Cutadapt (https://cutadapt.readthedocs.io/en/stable/guide.html) for trimming [alternative methods are reviewed in Conesa et al. (84)]. Some tools, like AfterQC (85), perform multiple operations (QC, filtering, trimming); also, while the steps mentioned above are performed on each sample separately, some tools are able to complete operations on multiple samples at once, such as SequenceImp (86), or to aggregate multiple outputs from a large number of samples in a unified report, such as MultiQC (http://multiqc.info).

### Alignment and detection of transcripts

After the initial processing and QC, alignment to the genome or transcriptome is performed in order to detect and quantify the transcripts in each cell. The choice of aligning to the genome or the transcriptome depends on the users' specific objectives. Nevertheless, in both cases, if spike-ins were used, their sequence must be added to the appropriate reference before alignment. Aligning to the transcriptome is usually faster but only feasible when dealing with well-annotated species such as *Homo sapiens* or *Mus musculus*, and even then, it cannot obviously discover new transcripts, although it grants the possibility to characterize isoforms. On the other hand, alignment to the entire genome can count both known and unknown transcripts but requires “splicing aware” tools to correctly detect splicing junctions. STAR is a widely used program for transcripts alignment to the genome that allows the discovery of non-canonical splicing patterns and chimeric transcripts (87).

UMIs can be directly used to count transcripts when alignment to the genome is performed (Figure 3); otherwise, expression levels need to be quantified by counting the reads aligned to each gene (HTSeq or featureCounts) (88, 89). Additional feature counting is not required if full length transcripts were sequenced allowing to use pseudo-aligners, such as Salmon (90) and Kallisto (91), that have lower requirements in terms of computational resources, resulting in a faster alignment, and are more robust to sequencing errors.

### Filtering

Once sequencing data have been aligned to the reference and transcripts are counted, several filtering steps are necessary to remove outliers or low-quality data, which might include wells containing zero or multiple cells, or broken or dead cells. The scater package (92) for the R environment (https://www.R-project.org/) is a useful tool to handle single cell data, perform filtering steps and preliminary exploration on the data. Some per-sample metrics are used as measures of sample quality, most notably the number of reads mapped (also referred to as library size or sequencing depth), the number of features (genes or transcripts) detected, and the percentage of reads mapping to mitochondrial genes or to spike-ins (when these are included in the design). Mapped reads and detected features should produce near-normal distributions, but the absolute values depend on the protocol used. Increased mitochondrial RNA content is usually associated with dead cells, however, cells with increased mitochondrial content and activity, as cardiac muscle cells, might physiologically produce a higher percentage of reads mapping to mitochondrial genes. The distribution of these metrics alone allows to spot outliers; however, more sophisticated approaches that considers both technical and biological features (such as genes upregulated or downregulated in broken cells) are available (93). Importantly, filtering data originating from doublets or aggregates of more cells can be coupled with demultiplexing, as done by demuxlet (94), or use methods based on the read counts, such as cellity, DoubletDecon (95), DoubletFinder (96), and DoubletDetection (https://github.com/JonathanShor/DoubletDetection).

### Normalization and removal of confounding effects

Normalization is required to remove systematic technical variation and ensure accurate inferences of expression levels. Theoretically, external spike-ins, which are added in the same amount to each cell, could be used to detect artificial variation introduced by the procedure (97). Although this approach is reliable in some instances (98), inconsistencies among replicates have prompted the need for alternative methods, also needed when no spike-ins are used.

Normalization designed for bulk RNA-seq relies on endogenous transcripts, global scaling, and scaling factors such as reads per million (RPM) or reads per kilobase per million (RPKM) (99) performs inconsistently for single cell data analysis, particularly when sequencing depth is low. Moreover, global scaling does not accommodate for transcripts specific biases deriving from systematic variation in the relationship between read counts and sequencing depth in scRNA-seq. To overcome this limitation and avoid over- or under-correction of some transcripts, SCnorm (100) splits transcripts in classes based on the dependence between counts and sequencing depth, then estimates a scaling factor for each class and normalizes the classes separately.

Besides library size, batch effects arising from sample processing should be minimized with an appropriate experimental design, for instance introducing replicates or with a balanced design including different conditions/individuals on the same plate or sequencing lane. Notably, to integrate data sets that are produced in different laboratories and at different times, reduction of batch effects is critical to avoid compromising the interpretation of data. Dimensionality reduction methods such as principal component analysis (PCA) can help detecting batch effects. Scater can plot PCAs and estimate the correlation between PCs and technical variables such as batch, plate, or replicate, as well as detected features or library size (92).

Once confounding factors have been identified, these need to be removed, for instance using control genes (or spike-ins) or negative control samples for which the covariates of interest are constant via the RUVSeq package (101), or by leveraging a subset of the population shared between batches (102).

While it might hold biological interest *per se*, cell cycle stage is another possible confounding factor: individual cells are typically not synchronized, and fluctuations in gene expressions across the cell cycle might mask or inflate cell heterogeneity, with an effect not limited to known cell cycle genes (103). Cell cycle stage can be treated as a latent variable, and accordingly modeled and corrected for, improving the identification of cell populations and the identification of correlated patterns of expressions across cells (103). A different approach, implemented in the cyclone tool, uses a set of six predictors to explicitly assign cell cycle stage to each cell, for G1, S, and G2/M phases (104).

### Post-normalization processing

Further post-normalization processing is required to tackle the main technical features of single cell RNA sequencing: noise and sparsity (abundance of missing values). Variability in the efficiency of RNA capture, retrotranscription, and sequencing can introduce technical noise which might mask the biological signal of interest. Modeling the contribution of noise allows to remove it or use it to select genes which are less affected by it. Endogenous oscillating genes, given their predictable nature, can be used to estimate technical noise (105). A different approach to model experimental noise and the dependence between noise and read count is to use external spike-ins that are added in fixed, identical quantity to each sample and should only be subject to technical variability (97, 106, 107).

To improve signal-to-noise ratio, feature selection can be applied. Although, this is not compulsory, it reduces computational times, and is required by some tools used in further steps. Feature selection can take advantage of spike-ins: after estimating the effect of noise using spike-ins, selection of genes with a significantly higher variability than that expected by the effect of noise alone, are used to study the heterogeneity across cells and make inferences on their biological relevance (106). Another approach to feature selection is based on the rate of dropouts, genes that are highly expressed in some cells and not detected in others, a less noisy measure than gene expression variability (108, 109).

The abundance of dropouts (or sparsity) is a relevant feature of single cell RNA-seq data, and can be alleviated by imputing missing values using the information from co-expressed genes, or from similar cells, with several tools developed to recover the “true” expression signal (110, 111). Interestingly, a recent comparison of several imputation methods (112) concludes that no imputation method outperforms all the others in every situation.

### Clustering: the first step for a solid biological analysis

One of the key aims of single cell approaches is the identification of different cell populations. When the biological background is well-known, checking expression profiles of known marker genes will help retrieve known cell types. This approach requires the application of dimensionality reduction methods such as PCA or t-distributed stochastic neighbor embedding (tSNE) to the refined gene expression counts, in order to identify and visualize subgroups. However, PCA usually requires assumptions that do not hold with scRNA-Seq analyses, and tSNE is better suited for visualization purposes, but is not appropriate for dimensionality reduction in preparation of further analyses (113). An alternative dimensionality reduction approach, specifically tailored to the high proportion of dropouts in single cell RNA-sequencing, is the zero-inflated factor analysis (ZIFA) which specifically models the dropout probability (114).

The identification of unknown and possibly rare subpopulation without prior knowledge, on the other hand, requires unsupervised approaches. Generally speaking, these methods compute one or more measures of “distance” between samples (115), then group cells based on their differences or similarities, as described by these distances. Different strategies can then be employed: hierarchical clustering, for instance, organizes cells in a tree structure according to their “distance,” following an agglomerative or divisive algorithm. K-means approaches, on the other hand, assign cells randomly to a predetermined number *k* of clusters, then adjust the assignment iteratively reassigning cells to the closest cluster, until no more reassignment is obtained. It is important to note that all clustering methods require some *a priori* decision from the observer, be it the height where the tree should be cut in hierarchical clustering, the *k* number of clusters or neighbors in k-means, the density parameters in density-based clustering, or the number of neighbors for each cell when constructing a graph. This depends on how fine or coarse is the cell types' definition required to address the biological question. This can switch from a smaller number of well-differentiated clusters, representing cell types or abundant populations, or a larger number of clusters reflecting small differences and highlighting rare sub-populations, and fine changes in cell states (109).

### Biological analysis beyond clustering and toward functional inferences

The ultimate goal of identifying cell subgroups is the biological inference that can be drawn from it. This typically consists in identifying genes which are differentially expressed (DE) across cell populations, and notably pointing to marker genes specifically associated with one of the identified clusters. Some clustering tools also perform these tasks; for instance, SC3 (116) automatically determines DE genes and marker genes using non-parametric statistics. Rather than simply testing for different expression levels, methods such as SCDE (117), explicitly model, and keep into account, the probability of undetected genes.

Well-beyond this initial descriptive step a number of functional inferences can be generated on dynamic processes, including intracellular gene regulatory networks but also on intercellular mechanisms. Even when single cell experiments only take a snapshot of a process, the cells can theoretically be reordered along a “trajectory,” sometimes called “pseudotime,” where each cell represents a different state of the progression (118), allowing the identification of transcriptional dynamics and differentially expressed genes across cell types/states. Trajectory inference methods include a dimensionality reduction stage and, possibly, clustering followed by a trajectory modeling stage (119). Some methods, such as, TSCAN (120), infer a linear trajectory, while others, including Monocle, its new implementation Monocle2 (121), and K-Branches (122), reconstruct a branching trajectory. Not all methods aim to a unidirectional trajectory: Oscope (105), for instance, infers oscillatory trajectories, aiming to identify oscillating genes and reproduce periodic signaling pathways such as those taking place in embryonic development (123).

Dimensionality reduction is a key step of trajectory inference, and indeed a family of tools for dimensionality reduction, diffusion maps (124), inherently reconstruct a trajectory. Importantly, diffusion maps have been adapted to single cell RNA-seq by accounting for uncertainties and missing values (125), as implemented in the R package destiny (126). These are used to identify the diffusion-space direction which most probably represents true biological effects. An exhaustive list of pseudotime ordering algorithms is found at https://github.com/agitter/single-cell-pseudotime.

Systematic computational analysis of the molecular interrelationships between cells in single cell populations studied can be performed by predicting and visualizing cell-cell communication based on the creation of a repository of curated ligands and receptors, which draws on existing resources and the literature (26, 127) using the biomaRt R package (128).

The acquisition of transcriptomic data at the single cell level has enormous potential to infer gene regulatory networks (GRNs). These can be viewed a set of genes active in a specific moment or condition, and the set of transcription factors activating them (52). A first class of approaches, such as BTR (129) and LEAP (130), requires cells to be ordered across different time points or in pseudotime trajectories, and reconstructs a network as a sequence of activations of transcription factors, aiming to predict the effect of perturbations of such sequence. A second class of methods, such as SCENIC (131), uses cells from steady states, leveraging co-expression patterns and representing GRNs as global networks, leading to the identification of key regulators for different cell states even on snapshot-type datasets and independently of any temporal or pseudotemporal ordering.

Recently, the remarkable development of technologies like spatial transcriptomics, a technology used to spatially resolve RNA-seq data (132), or FISH-based techniques (133), requires the integration of spatial information with mRNA profiling of single cells. SpatialDE allows to fully integrate spatial information and the detection of differential expression (134), enabling the identification of linear or periodic expression patterns, clustering genes based on their spatial expression patterns, and describing tissues on the basis of marker genes' expression patterns (132).

In summary, the computational approaches available allow for unprecedented biological functional inferences and highlight the need for the development of teams including wet-lab biologists, computational biologists and clinicians to exploit the technologies and data available in understanding cardiac disease.

Before moving to the review of scRNA-seq applications to cardiac studies, a short overview of single cell qRT-PCR as a rapid platform to study the expression of tens of genes in hundreds of single cells is discussed. Methods for cell selection are similar to the ones reported above for scRNA-seq. The major advantage of single cell qRT-PCR is the ability to go from cell isolation to analyzed results in less than a working day; on the other hand, scRNA-seq methods tend to be considerably more expensive and laborious, also considering the much higher requirements for data storage and computational power for data analysis. (25, 135). In deciding which approach to use, one shold consider that single cell qRT-PCR is useful to analyse up to a few hundreds cells, while scRNA-seq allows to survey up to tens of thousands of cells. Moreover, single cell qRT-PCR is tipically used to test the expression of tens of pre-selected genes, while scRNA-seq detects, in theory, the whole transcriptome, with the general limitation of sequencing depth. Albeit, full-length sequencing methods such as Smart-seq2 are potentially able to discriminate between different isoforms, most of the methods currently used, such as 10X Genomics, only produce a count of transcripts sequencing UMIs, with no isoform discrimination.

A series of qPCR assays were used to compare gene expression profile of different cells from cardiac lineages (cardiac progenitors, smooth muscle cells, CMs, endothelial cells, fibroblasts) with embryonic stem cells (mESCs), using the detected profiles to later classify unselected E10.5 cells and finely compare *in vitro* mESC- and *in vivo* embryo-derived cardiac cells. These studies highlighted a propensity of Nkx2-5+ progenitors from *in vitro* mESC to become smooth muscle cells or CMs, while the ones from embryos had preferential differentiation toward CMs or endothelial cells (24).

Data have also been obtained from freshly isolated cardiac cells of the adult heart. Single cell gene expression analysis for a selected number of genes was proven to critically augment the definition of adult cardiac progenitors, candidate cell therapeutic products and putative *in situ* cell targets for reparative and/or regenerative purposes in myocardial ischemia (136). Indeed, the combination of FACS, clonogenicity assays and single cell qRT-PCR for about 40 genes on a few hundred of cells permitted the refinement of adult cardiac Sca1+ cells into four discrete populations. Sca1+ CD31+ PDGFRa- cells were found to be enriched for endothelial lineage markers, while Sca1+ CD31- PDGFRa+ cells precisely track a cardiogenic molecular signature (25). Importantly, co-expression of Sca1 and PDGFRa with the side population phenotype, that *per se* enriches for cardiogenic and cardioprotective features, allowed to identify a cardiac subpopulation that is over 30% clonogenic (25).

Interestingly, single cell gene expression was also used to study the phenotype of human induced pluripotent stem cell (iPSC) derived CMs, an alternative cell therapeutic product for myocardial infarction, in a mouse model (137). Combining molecular imagining techniques, microfluidic single cell profiling and laser capture microdissection, iPSC-CMs were shown to contribute to heart repair via an early protective paracrine effect on the ischemic microenvironment.

As quantitative RT-PCR still represents the gold standard for gene expression analysis, its application at the single cell level is a rapid and precise tool to be used alone or in conjunction with single cell RNA-sequencing, as a preliminary exploration for selection purposes or as a validation tool (24, 138).

## Single cell state-of-the-heart

### Single cell technologies provide a novel perspective and depth of resolution to study the development of the cardiac system

As for many other organs, the first single cell ‘omics studies on the heart were used to characterize the cardiac cellular landscape during development. Within this space, the advantages of single cell transcriptomics analysis include an increased bandwidth to capture rare cell types like stem and progenitor cells, and the capacity to study cellular states at the moment when fate decisions are executed, overcoming limitations of single cell transplantation experiments or lineage tracing that enable to pick events only once they have occurred.

Characterization of cardiac progenitors in a very narrow window of time during early development (embryos of early allantoic bud, late allantoic bud, early head fold) was possible using a combination of micromanipulation for cell selection and single cell qRT-PCR for validation of cells identity, followed by scRNA-seq (43). The integration of scRNA-seq data and subsequent validation steps, including lineage tracing experiments, revealed a rapid dynamic shift of the expression profile of *Tbx5*+ cardiac progenitors with diverging patterns between first and second heart fields CMs' precursors.

A more complex and comprehensive approach was applied to study the mesodermal lineage diversification from early gastrulation (E6.5) to the generation of primitive red blood cells at E7.75 (51).The combination of FACS for single cell capture and Smart-seq2 enabled the profiling of cellular populations that was previously challenged by the limited cellular material available. By sequencing ~1,200 cells, including epiblast cells sorted based on viability, Flk1+ cardiac mesoderm cells and CD41+ cells that subsequently appear during blood development (139), it was possible to define for the first time the transcriptome of single cells from the developing primitive streak, and a limited number of visceral endoderm and extra- embryonic ectoderm cells, primitive streak, neural plate and head fold (51). Unsupervised clustering isolated ten clusters and correlated marker genes allowing functional inferences and identification of endothelium, blood progenitors, primitive erythrocytes or their anatomical identity (visceral endoderm, extra-embryonic ectoderm, epiblast, early mesodermal progenitors, posterior, allantoic, and pharyngeal mesoderm). Here, diffusion maps were used to make inferences on the transcriptional program underlying primitive erythropoiesis. Further, given the fundamental role of the transcription factor Tal1 in the development of all blood cells, single mesodermal Flk1+ cells from *Tal1* mutant embryos were profiled (51). The combination of loss of function studies with single cell transcriptomic analysis demonstrated, in contrast to previous retrospective knock-down and epigenetic studies (140, 141), that Tal1 promotes hemogenic differentiation of endothelial cells but does not immediately drive a cardiac fate. The difference in time of sampling (141) could justify the differences seen between a precise snap-shot of single cells transcriptomic profiling (51) vs. potentially confounding retrospective studies in bulk (140, 141). It is foreseeable that performing transcriptomic and epigenomic assays (142) in parallel to profile a high number of single cells in a fine time course will further clarify the mechanisms of early mammalian development and more specifically the fate of Flk1+ cells.

From the next stages of cardiac development (E8.5 to 10.5), profiling of 2,233 single cells using a microfluidics approach (Fluidigm, C1 single cell Auto Prep System) and Smart-seq2, combined with unsupervised and supervised clustering allowed the reconstruction of the precise spatial origins of cardiac cells solely from their transcriptional profiles (138). Interestingly, a distilled set of 65 genes was validated and found sufficient to predict location of cardiomyocytes using a multiplex qRT-PCR-based approach (24, 138).

The dynamic transcriptional programs unfolding from midgestion (E9.5) to adulthood (P21) were studied using spatiotemporal RNA-seq analyses of single cells isolated from mouse hearts and captured using microfluidics, to delineate lineage specific transcriptional regulation, including CM maturation, during heart development (143). Classification of different cell types at the single cell level with spatiotemporal resolution allowed the definition of time and chamber lineage-specific gene programs underlying normal cardiac development. Further post-natal maturation occurred by P21 with downregulation of calmodulin-interacting proteins, Bex1 and Bex4, previously shown to promote muscle regeneration (144). Albeit, one should consider the potential bias introduced by size limits inherent to the microfluidics system used to select large mature CMs, these datasets are useful to define precise developmental stages of human and mouse embryonic stem cells-derived CMs, prompting the utility for characterization of induced pluripotent stem cells-derived CMs that are becoming the gold standard in cardiac drug discovery (27, 145). Notably, analysis of single cells from Nkx2.5^+/−^ murine hearts were used as a model for congenital heart disease and allowed to define lineage-specific maturation defects, including expected changes in the CM lineage but also in the endothelial lineage compartment. This supports an instructive role of CMs on endocardium development and predicting underlying mechanisms associated with human heart malformations.

The experiments herein described prove the advantage of using single cell transcriptomics in enabling the study of cellular states at the moment when fate decisions are executed, contributing to the precise deconvolution of lineage decisions previously masked by bulk analysis and, especially in early stages, difficult to study given the paucity of cells available.

### Adult heart and cardiovascular disease models from a single cell perspective

Compared to other areas of research—until very recently—the cardiac field has infrequently taken advantage of single cell technologies to study adult heart cells' gene expression and, even less, for epigenetic studies. Although, cells of the adult heart are not readily accessible, a number of protocols have been historically described to dissociate single CMs and single stromal cells. If some skepticism can be raised by the fact that isolation protocols are lengthy and potential side effects on gene expression are a deterrent, the use of whole tissue as comparison and validation experiments obviates any doubt. The only technical impediment remains the size of adult mature CMs (~150 μm in length) that limits the use of microfluidic apparatuses with upper size limits usually around 50 μM (except for ICELL8).

Albeit the regenerative capacity of the adult heart is meager, there is some evidence that induction of CM proliferation could be a targetable mechanism to induce myocardial self-renewal and repair after injury (146, 147). The proliferative potential, especially the capacity to undergo cytokinesis of CMs in physiological conditions as well as in response to stress, remains unclear. Confounding factors could be the methods of analysis but, most importantly, also the absence or presence of external stimuli that can enhance CMs proliferation and self-renewal (146, 148–150). Recently, to understand the proliferative capacity of CMs, single nuclear RNA-seq was performed in single CMs from healthy and diseased hearts, including pressure overload failing murine hearts and human dilated cardiomyopathy failing hearts (151). Heterogeneity of the myocardial environment in response to stress was uncovered (151). Through weighted gene co-expression network analysis, several subgroups of CMs were characterized, including ones with a gene signature indicative of dedifferentiation and cell cycle entry. Further, two long intergenic non-coding RNAs, *Gas5* and *Sghrt*, were found in highly interconnected nodal hubs of gene regulatory networks and, subsequently, confirmed as essential regulator of CMs' cell cycle entry and de-differentiation programmes (151). These data are a first and encouraging step toward answering additional fundamental questions: the functional implications of specific gene expression patterns in the various CMs subtypes; which subtypes are elicited or depressed in diverse pathological conditions, including early stages of dilated cardiomyopathies, hypertrophic cardiomyopathies and ischemic disease; the physical localization of CMs subpopulations and how the cellular demographics change in response to stress; finally the concasual relationships with neighboring non-CMs.

A complementary study focused on the cardiac stromal compartment and used a 10X Genomics platform to capture and sequence just over 10,000 non-myocyte cells (26). With a mean of 1,900 genes detected/cell, an expected overall complexity in the composition of the cardiac non-myocyte cellular compartment was revealed and a few additional markers and subpopulations were identified in the fibroblasts compartment and in the lymphocytes and myeloid lineages (26). Interestingly, clustering patterns for female and male cells were largely overlapping but within individual cell types, small levels of sexual dimorphism was detected for selected genes' expression, implying a diverse predisposition of the cardiac stroma response to stress (26).

A further level of complexity was obtained by comparing scRNA-seq results from healthy vs. post-ischemia mouse hearts using the SORT-seq protocol, which is essentially an integration of FACS for single cell capturing and high-throughput multiplexed linear amplification by CEL-seq2 (29, 48). In this study, cell capturing has two limitations: (1) large mature CMs have dimensions above the size of the sorting nozzle, (2) the utilized flow cytometry gates were biased toward large live cells (29). Thus, the representation of the cell types was biased, with 71% of CMs vs. the 30% expected, plus a putative selection of myocytes subtypes due the upper size limits of the sorter outlet (29). Nevertheless, all main cardiac cell types were recognized as seen using differential gene expression analysis and clustering methods. In addition, identification of new putative markers including ones for specific subpopulations were pinpointed. Amongst these, *Ckap4* was defined as a novel marker for activated fibroblasts post-ischemia in mouse, and subsequently confirmed as a marker in human ischemic hearts. CKAP4 function remains to be clarified, although knock-down experiments suggested it controls the expression of activated-fibroblasts genes. Interestingly, in single CMs' sequencing, up to 84% of reads were coming from mitochondrial genes (29). This is surely in part dependent on the high enrichment of mitochondria in CMs; however, while enrichment of mitochondrial related gene categories are amongst the features characteristic for low quality cells in a number of independent cellular models, this is not the case for myocytes (93). Thus, it could be necessary to establish a specific set of features to determine the quality of single heart myocytes based on their gene expression.

Single cell transcriptomics analysis was used to characterize the changes occurring at the single cell level in a mouse model of cardiac fibrosis (28). Co-expression analysis inherent in single cell transcriptomics studies allowed to highlight that both IL-11 and its receptor IL11RA are expressed in activated fibroblasts, the latter defined by a transcriptional profile typical of TGFβ stimulated cells and by induction of extracellular matrix encoding genes (28).

Tissue regeneration using exogenous products, including iPS-CMs, is a highly sought-after therapeutic approach but much remains to be understood regarding the level of differentiation, purity, genomic, and phenotypic stability of induced CMs especially because, until recently, gene expression studies were almost exclusively performed on bulk populations. Reprogramming fibroblasts into induced cardiomyocytes (iCMs) using combinations of cardiogenic transcription factors (152–154) offers an interesting alternative to the use of iPS-CMs to generate newly formed CMs *in vitro* and then used as cell therapy, but also for *in vivo* direct reprograming. To study and characterize the non-conventional differentiation mechanisms driving cell conversion to iCMs, single cell approaches are a suitable option allowing to unpick the heterogeneity of conversion events and the level of differentiation in single cells. Single cell transcriptomics analysis of the early stages during the reprogramming of mouse fibroblasts into iCMs uncovered a heterogeneous group of cells differentiating at unsynchronized pace (155). The definition of different cell clusters based on dimensionality reduction and unsupervised clustering algorithms was the critical step to make functional inferences. Some of these were anticipated, like the reconstruction of differentiation trajectories, the correlation between the expression of each reprogramming factor and the progress of individual cells through the reprogramming process, and the discovery of new surface markers for iCMs (155). Among other discoveries, the downregulation of factors involved in mRNA processing and splicing was found to be critical in reprogramming; interestingly, a specific splicing factor, Ptbp1, was identified and subsequently functionally validated to be a critical barrier for the acquisition of CM-specific splicing patterns in fibroblasts (155). Thus, in this settings scRNA-seq enabled the identification of targets to enhance reprogramming efficiency and new markers for the prospective isolation of iCMs but also demonstrated the immense utility of these methods in studying further models of programming and reprogramming.

An *in vitro* system was used to study the dedifferentiation potential of adult CMs to cardiac progenitor-like cells, using a genetic cell fate mapping system plus single cells transcriptome and further methylome analysis (156, 157). The authors reported a correlation between hyper-methylation of promotor regions and suppression of cardiac specific genes related with maturation, while cell cycle and stemness genes were upregulated suggesting that CMs' de-differentiation depends on epigenomic regulation of the cells transformed into progenitors. Nevertheless, this study relies on very few single cells and confirmation of the specific gene expression regulation via epigenetic modulation would benefit from analysis of the transcriptome and methylome from the same single cell.

Within the cardiac regenerative medicine space, c-kit+ adult cardiac progenitors have been proposed as a cell product albeit controversial data were reported (158). Recently, single freshly isolated adult c-kit+ CPCs were compared with short-term expanded cultures by 10X Genomics and SmartSeq2, in parallel with the meta-analysis of multiple scRNA-Seq datasets, finding substantial transcriptome alterations in the *in vitro* expanded cells (159). This increase in the transcriptome diversity included the induction of thousands of genes related to cell cycle and metabolism, and loss of expression of identity genes, suggesting a marked change in functional characteristics of *in vitro* expanded cells, with important implications to consider when developing cell therapies than involve previous *in vitro* expansion.

Altogether, these initial explorative studies both in healthy and disease hearts demonstrate how single cell transcriptomics alone has already contributed to uncover novel cell types, further our understating of candidate cell products for cardiac cell therapies and most importantly discover novel functional pathways regulating heart function.

### Predicting achievable outcomes and influencing future clinical applications

Thanks to the immense technological advancements of high-throughput single cell gene expression analysis and the high-resolution achieved with scRNA-seq, we can now obtain information on prevalence, heterogeneity and co-expression at the individual cell level for virtually the entire transcriptome. This enables us to get a new perspective on cellular demographics of the heart and improve intracellular and intercellular pathways definition, otherwise unthinkable with bulk analysis and in circumstances where cellular material available is limited. The almost immensurable amount of data being produced requires tight integration and interaction of biological and clinical experts with computational biologists starting from the initial experimental design. In fact, a dynamic and continued assessment of data generation and results is necessary to reach solid conclusions of any single cell ‘omic study.

The complete exploitation of the value of single cell transcriptomic results comes from the integration and validation of these data by orthogonal methods. Validation of the expression of subsets of genes can be performed by standard techniques with emphasis on single cell resolution approaches ranging from gene expression validation with or without spatial resolution (e.g., single molecule *in situ* hybridization, single cell qRT-PCR, respectively), to testing the expression of the proteins encoded by the genes of interest, by flow cytometry that allows a higher bandwidth for multiplexing or immunostaining to acquire localization information. Of note, spatial transcriptomics was developed in order to retain spatial information related to the transcriptome of isolated single cells and was elected Method of the Year 2017 by Nature Methods (160). The protocol requires tissue sections that are then divided in a mosaic of tiles of ~100 μm of diameter, and uses spatial barcodes permitting the localization of specific cells and their transcripts by overlaying haematoxylin & eosin stained sections onto microarrays spotted with the barcoded topographies (132, 161). The first reported application of single cell RNA-seq and spatial transcriptomics was to identify *in vivo* genes relevant to endocardial epithelial-to-mesenchymal transformation (162). More recently, spatial resolution proved to be essential in the identification of SOX9 as a key regulator of cardiac fibrosis in a mouse model of ischemic injury and injured human heart (163). Critically, rather than just allowing to localize the cell types identified by scRNA-seq, spatial transcriptomics will expedite the reconstruction of the dynamic architecture of the heart (134). Indeed, changes of gene expression and especially of transcriptomic profiles can depend on the reciprocal interactions with adjacent cells, the migration to a specific tissue site of cells' subsets and by location-specific variation of cell “states.” Thus, starting from a single cells and functional micro-niches of few cells, one can envision to reconstruct the changes occurring in the cellular landscape of whole tissues and organs in 3D during specific processes including development, aging, and disease (134).

**Figure 4:**
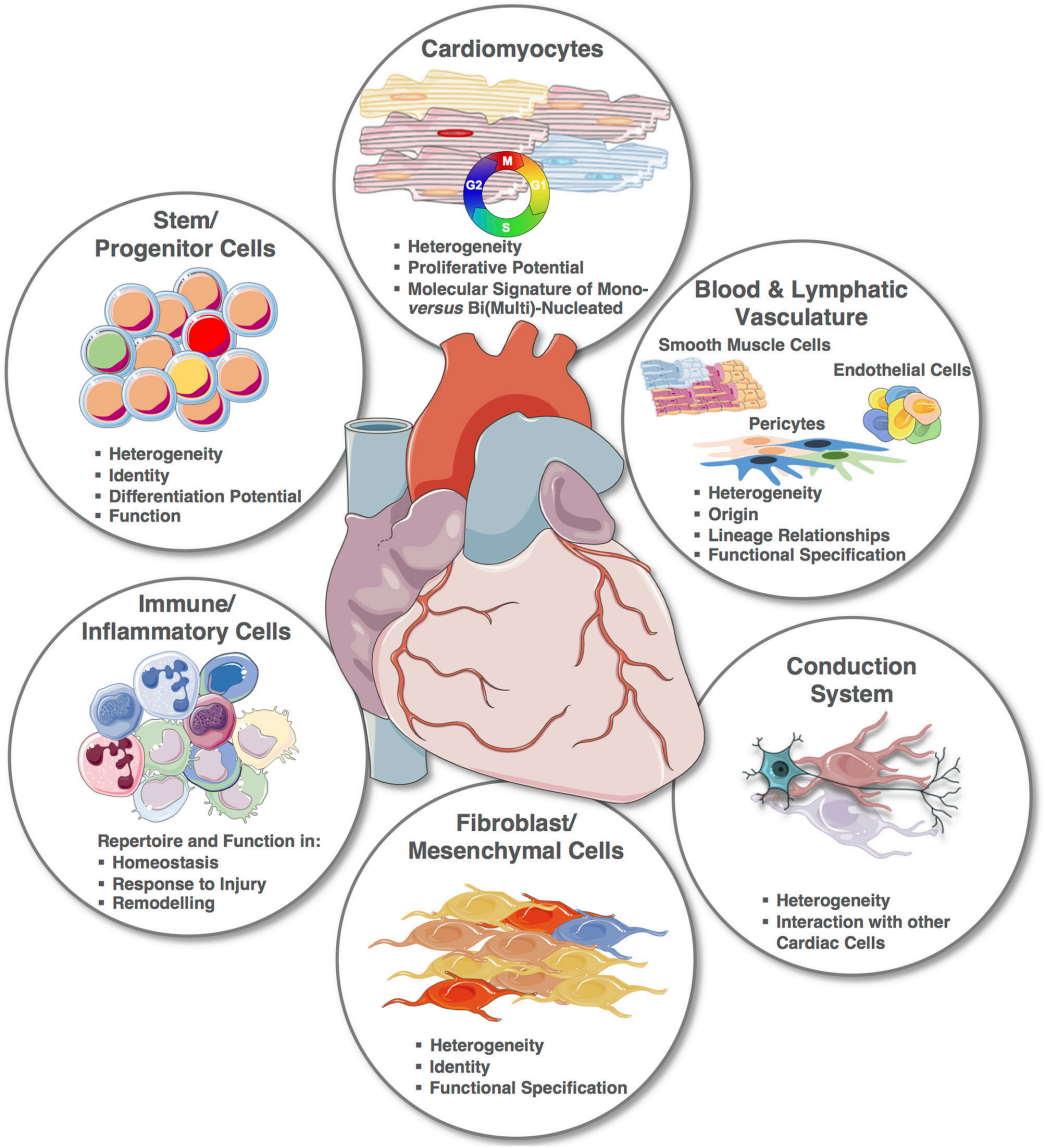
The cellular cardiac landscape: expected deliverables and improvement using single cell and spatial transcriptomics. Single elements used to construct the figure were taken from https://smart.servier.com/ and minor modifications (e.g., color) were applied.

Despite the recent explorative studies, the cellular cardiac landscape, especially human, remains largely unknown (Figure 4). Most significantly, the integration of single cell transcriptomic and spatial transcriptomic provides the platform to reconstruct the cellular demographics of the heart with spatial resolution. For smaller animals, this will require a shorter period of time, and although the reconstruction of the entire human cardiac cellular landscape in 3D will require many iterations, every step will provide new strategic information. For example, cardiac muscle scRNA-seq will alone contribute to the clarification of CMs' heterogeneities within microniches, on the epicardial vs. endocardial fronts; as well as chamber specificities that remain largely unknown. Notably, one of the therapeutic targets for cardiac regeneration is CMs' proliferation, but for clinical translation we still need to: (1) clarify the true endogenous and spontaneous capacity of human CMs to re-enter cell cycle and undergo mitosis since results in mouse remain controversial and (2) define the stimuli that can trigger or improve their proliferation. Importantly, transcriptomic analysis of single mononucleated vs. binucleated CMs and comparison with single nuclei transcriptomics will provide inferences on their proliferative and differentiation status (146, 148–151, 164).

The identity and role of cardiac progenitors in the adult heart remains elusive with a dozen different identifiers already described, and their relationship and function still unclear (136). It is expected that an unbiased ‘omic approach will allow to define their molecular signatures and likely deconvolute their ontogeny and function in the healthy and diseased heart.

The heterogeneity of vascular cells across organs and within the same organ or system has been hypothesized for a long time but the advancements in uncovering of subpopulations with distinct functions has been slow because of bulk analysis and largely limited to hypothesis driven studies that can focus on one gene/pathway at the time. The availability of single cell transcriptomics and spatial transcriptomics is providing the right tools to study endothelial cells, smooth muscle cells and pericytes' subtypes and functional changes across vessels of different diameter and function. A recent study proved feasibility and produced outstanding results by sequencing the vascular cells of the mouse brain (165) uncovering the transcriptional basis of the gradual phenotypic change along the arteriovenous axis and revealing unpredicted differences in the cells' molecular signatures with a seamless continuum for endothelial cells vs. a punctuated pattern for mural ones (165). Importantly, brain specific features were highlighted for pericytes in comparison to the lung. These important data provide the evidence for a higher organizational structure of the vasculature, providing a healthy reference for subsequent disease studies, but also provides an invaluable hypothesis-generating data source.

Although initial scRNA-seq studies in the adult heart have already contributed to highlighting the role of specific genes in the fibrotic response after TGFβ1 stimuli (28) and ischemic injury (29), much remains to be learnt about the identity of fibroblasts, their function, heterogeneity, anatomical specificity and role in cardiac homeostasis and disease.

In recent years, a number of studies have investigated the adaptive and innate immune response following cardiac injury beyond the response to microbial causes, how these affect repair and regeneration and their potential manipulation as therapeutic targets [as reviewed in Prabhu and Frangogiannis (166), Sattler et al. (167), Toldo and Abbate (168), and Wysoczynski et al. (169)]. Single cell transcriptomics focusing on immune cells will provide an invaluable support to decode higher organizational networks triggered in cardiac disease and in physiological conditions. Finally, single cell transcriptomics will also contribute to our understanding on the conductive system of the heart, both in unbiased studies and targeted analysis.

Studying the developing heart at the single cell level has an inherent huge power, not only to further our understanding of the perfect orchestration needed for cells to go from a primordial linear tube to a four-chamber heart and uncover maps and trajectories of cellular differentiation but also, importantly, to discover how lineage-specific gene programs are altered in congenital heart disease (24, 27, 43, 51, 138). This implies a need to study and build a developing atlas of the healthy developing heart in animal models but most importantly in human, and use this as reference to study and understand the genesis of cardiac malformations within single cells, and between them.

Two studies have generated independent atlases for mouse models, providing an initial compendium of single cell transcriptome profiling representing a new resource for cell biology (170, 171). Providing gene expression data from 20 anatomical locations, the atlases allow the comparison across tissues of cell types present in every tissue such as immune cells and endothelial cells enabling hypothesis generation. Strikingly the Human Cell Atlas (HCA) initiative has triggered widespread excitement with its ambitious goals aiming to describe and define the complete cellular landscape of the human body (https://www.humancellatlas.org/) (172, 173). This will be achieved by mapping cells of the human body using a combination of single cell and spatial transcriptomics, resulting in datasets that go beyond descriptive features and will guide functional studies toward the identification of previously unpredictable subtleties of disease. Effectively, the HCA plans to integrate leading-edge technologies to: (1) defining the histological and anatomical position of newly identified cell types/states; (2) outlining lineage decisions with topographic and functional insights; (3) recapitulating precise and specific cell-to-cell interactions including autocrine and paracrine pathways.

Urgent clinical questions, that would immediately benefit from an integration of single cell transcriptome studies and spatially resolved gene expression profiling, include the definition of the changes occurring within the cellular landscape of the border zone following myocardial ischemia; the deconvolution of atherosclerotic plaque's single cell transcriptomic profiles. Inferences in ethiopathogenetic mechanisms that are invisible using standard low resolution and bulk techniques include proliferation, differentiation, survival as well as ligand/receptor pairs to infer functional networks. Additionally, transcriptomic profiling of single cells will undoubtedly enable the definition of novel biomarkers, including parameters such as frequency and pattern of gene expression within tissues and/or specific cell types.

Altogether, these new technologies will not only contribute to address hypothesis-driven questions but, most importantly, will broaden our perspective, providing hypothesis generating platforms to further our understanding on heart function in health and disease. The auspicable future of cardiac disease monitoring is based on low-risk, minimally invasive and disruptive approaches, where multi-‘omics single cell approaches will play a leading role.

## Author contributions

All authors listed have made a substantial, direct and intellectual contribution to the work, and approved it for publication.

### Conflict of interest statement

The authors declare that the research was conducted in the absence of any commercial or financial relationships that could be construed as a potential conflict of interest. The handling Editor declared a shared affiliation, though no other collaboration, with several of the authors MN, AM, PC, SS at the time of the review.

## Abbreviations

CM: CardioMyocyte
DE: Differentially Expressed
ERCC: External RNA Controls Consortium
FACS: Fluorescence-Activated Cell Sorting
FISH: Fluorescent *In Situ* Hybridization
GRN: Gene Regulatory Network
HCA: Human Cell Atlas
iCM: Induced Cardiomyocytes
iPSC: Induced Pluripotent Stem Cell
LCM: Laser Capture Microdissection
MARS-Seq: MAssively parallel RNA Single cell sequencing
mESC: Mouse Embryonic Stem Cell
MSC: Mesenchymal Stem Cell
PCA: Principal Component Analysis
qRT-PCR: Quantitative Real Time PCR
RPKM: Reads Per Kilobase per Million
RPM: Reads Per Million
SCRB-seq: Single-Cell RNA Barcoding and sequencing
scRNA-seq: Single Cell RNA-sequencing
Smart-Seq: Switch Mechanism At the 5’ end of RNA Templates sequencing
snRNA-seq: Single Nuclei RNA-sequencing
tSNE: T-distributed Stochastic Neighbor Embedding
UMI: Unique Molecular Identifie
ZIFA: Zero-Inflated Factor Analysis.
